# Systematic Review and Meta-Analysis of the Utility of Circular RNAs as Biomarkers of Hepatocellular Carcinoma

**DOI:** 10.1155/2019/1684039

**Published:** 2019-05-02

**Authors:** Qingqin Hao, Yadi Han, Wei Xia, Qinghui Wang, Huizhong Qian

**Affiliations:** ^1^Department of Clinical Laboratory, Wuxi Red Cross Blood Center, Wuxi 214000, Jiangsu, China; ^2^Department of Clinical Laboratory, The Affiliated Cancer Hospital of Zhengzhou University and Henan Cancer Hospital, Zhengzhou 450008, Henan, China

## Abstract

Emerging studies have reported circRNAs were dysregulated in HCC. However, the clinical value of these circRNAs remains to be clarified. Herein, we aimed to comprehensively explore their association with the diagnosis, prognosis, and clinicopathological characteristics of HCC. PubMed, EMBASE, Web of Science, and Cochrane Library databases were comprehensively searched for eligible studies up to October 30, 2018. The diagnostic effect was evaluated by the pooled sensitivity, specificity, and other indexes. The pooled hazard ratio (HR) for overall survival (OS) and recurrence free survival (RFS) was calculated to assess the prognostic value. Ten studies on diagnosis, 12 on prognosis, and 23 on clinicopathology were identified from the databases. A total of 11 upregulated and 11 downregulated circRNAs showed an association with clinicopathological features of HCC. For the diagnosis analyses, the pooled sensitivity, specificity, positive likelihood ratio (PLR), negative likelihood ratio (NLR), and diagnostic odds ratio (DOR) of circRNAs for HCC were 0.74 (95%CI: 0.65-0.82) and 0.76 (95%CI: 0.70-0.81), 3.1 (95%CI: 2.5-3.8), 0.34 (95%CI: 0.25-0.47), and 9 (95%CI: 6-14), respectively. The area under SROC curve (AUC) was 0.81 (95% CI: 0.78–0.84), indicating moderate diagnostic accuracy. In stratified analyses, the diagnostic performance of circRNAs varied based on the source of control and specimen type. For the prognosis analyses, increased expression of upregulated circRNAs was associated with worse OS (HR: 3.67, 95%: 2.07-6.48), while high expression of downregulated circRNAs was associated with better OS (HR: 0.38, 95%: 0.30-0.48). In conclusion, this study reveals that circRNAs may serve as promising diagnostic and prognostic biomarkers for HCC. However, further investigations are still required to explore the clinical value of circRNAs.

## 1. Introduction

Hepatocellular carcinoma (HCC), a highly heterogeneous malignancy, is the second leading cause of cancer-related death worldwide [1, 2]. Although major progress has been achieved in prevention, detection, diagnosis, and treatment, a total of 782000 cases diagnosed and 746 000 deaths were estimated to occur in 2012 worldwide [3]. Currently, due to inefficient screening, HCC is often diagnosed at advanced stages; many patients therefore miss the optimal time for surgery [4, 5]. Furthermore, failure to identify patients at high risk of metastasis and recurrence has also resulted in an unsatisfactory prognosis of HCC patients. Therefore, there is an urgent need for more effective biomarkers for early detection and prognosis prediction of HCC.

Circular RNAs (circRNAs), a novel class of noncoding RNA, are generated by ‘backsplicing' of protein-coding mRNAs or linear noncoding RNA that join an upstream 3′ splice site and downstream 5′ splice site to form a covalently closed continuous loop [6]. They are highly stable, abundant and conserved, and involved in various physiological and pathological processes. However, the biological functions of most circRNAs are still unclear. Recently, emerging studies have revealed that aberrant circRNA expression has been observed in various cancers, such as colorectal cancer, breast cancer, gastric cancer, and HCC [7]. These circRNAs played crucial roles in the cancer-associated proliferation, angiogenesis, and metastasis and might be the key factors for cancer occurrence and development. To date, a series of articles have reported that circRNAs have great potential to serve as promising biomarkers for HCC. For instance, Qin et al. [8] found hsa_circ_0001649 was significantly downregulated in HCC. It might function in tumorigenesis and metastasis and could serve as a potential biomarker in the diagnosis of HCC (AUC = 0.63). In addition, cSMARCA5 could inhibit the proliferation and migration of HCC cells. The downregulation of cSMARCA5 was significantly correlated with aggressive characteristics and might serve as an independent risk factor for overall survival (OS) and recurrence free survival (RFS) in HCC patients [9].

However, due to the variances in study design, sample size, patient characteristic, and detection methods, the clinical value of circRNAs for HCC has not yet been fully elucidated. Previously, four published meta-analyses have reported the diagnostic and prognostic value of circRNAs for human cancers [10–13]; however, they included relatively few studies and patients and did not perform detailed analyses to explore the diagnostic value of circRNAs for HCC. Therefore, we performed this systematic review and meta-analysis to explore the relationship between aberrant cirRNAs expression and the diagnosis, prognosis, and clinicopathological characteristics of HCC.

## 2. Methods

### 2.1. Search Strategy

This meta-analysis was conducted according to the PRISMA guideline (Supplement File S1) [14]. We comprehensively searched for the relevant articles in PubMed, EMBASE, Web of Science, and Cochrane Library databases (up to October 30, 2018) assessing the potential clinical utility of circRNAs for HCC. A combination of the Medical Subject Headings (MeSH) and title/abstract words was used: (liver neoplasia or carcinoma or neoplasm or cancer or tumor) and (circular RNAs or circRNAs). We also manually searched relevant reviews and bibliographies of eligible articles to find out other potential studies.

### 2.2. Eligibility Criteria

The eligible studies should meet the following criteria: (1) the diagnosis of HCC was pathologically confirmed; (2) about evaluating the relationship between circRNAs and the diagnosis, prognosis, or clinicopathological characteristics of HCC; (3) for diagnosis, studies could supply sufficient information to construct the diagnostic 2 × 2 tables; and (4) for prognosis, HR (hazard ratio) and its 95% confidence interval (95% CI) can be extracted or calculated from the studies [15]. The exclusion criteria were as follows: (1) duplicate articles; (2) case reports, letters, reviews, editorials, and meeting abstracts; and (3) insufficient data.

### 2.3. Data Extraction and Quality Assessment

The following data were extracted: first author, year of publication, country, sample size, clinicopathological features, circRNAs profiles, altered expression, specimen type, detection method, reference gene, diagnostic data, follow-up period, outcomes, and HRs with its 95% CIs.

The quality of diagnostic studies was assessed with the Quality Assessment of Diagnostic Accuracy Studies 2 (QUADAS2). Meanwhile, the Newcastle-Ottawa Scale (NOS) was applied to assess the quality of prognostic studies [16], and a score ≥6 indicates high quality.

All these processes were performed independently by two reviewers (HQQ and HYD). Any discrepancies were resolved by consensus.

### 2.4. Statistical Analysis

All analyses were performed with the RevMan5.3 (version 1.4), STATA 12.0 (STATA Corporation, College Station, TX), Meta-Disc 1.4, and Engauge Digitizer 4.1 software. HRs with 95% CI were directly extracted from each study, if provided, or calculated according to the methods clarified by Tierney et al. [15]. A bivariate meta-analysis model was employed to calculate the pooled sensitivity, specificity, likelihood ratio (LR), diagnostic odds ratio (DOR), and HR with 95% CI, respectively. A summary receiver operator characteristic curve (SROC) was also established and corresponding AUCs with 95% CI were calculated [17, 18]. The Cochran-Q and Inconsistency index (*I*^2^) tests were applied to assess the heterogeneity among studies [19]. A* P* value (≤0.05) or* I*^2^ value (≥50%) indicated significant heterogeneity and the random-effects model was adopted. Otherwise, the fixed-effects model was used [20]. Spearman correlation coefficient was used to verify the threshold effect. To explore the sources of heterogeneity, we performed subgroup analysis and metaregression. Sensitivity analysis was further carried out to assess the robustness of the results. At last, publication bias was evaluated using Begg's funnel plot [21] and Deek's funnel plot, and* P *> 0.05 indicated no potential publication bias. All tests were two-sided and* P < *0.05 was regarded as statistically significant.

## 3. Results

### 3.1. Literature Selection

**Figure 1:**
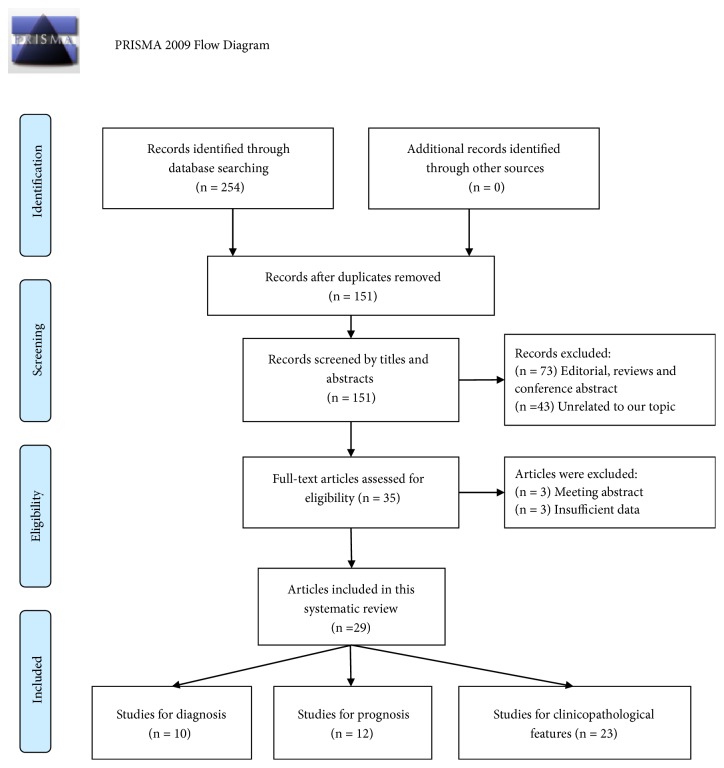
The flow diagram of the study selection process.

As described in Figure 1, a total of 254 articles were initially identified and 151 studies remained after excluding duplicate studies. By screening the titles and abstracts, 116 articles were further excluded because of editorial, reviews, conference abstracts, or irrelevant research topic. As a result, 35 remaining articles were for full-text review, and then 6 papers were excluded due to conference abstracts or insufficient data. Ultimately, 29 articles were included in this study, including 23 studies [8, 9, 22–42] on clinicopathological features, 10 on diagnosis [8, 22, 25–27, 31–33], 10 on OS [9, 26, 28, 34, 35, 38–40, 43, 44], and 2 on RFS [9, 45].

### 3.2. Correlation of circRNAs Expression with Clinicopathological Features

A total of 22 circRNAs from 23 articles showed an association with clinicopathological features of HCC. As summarized in Table 1, hsa_circ_0128298, circRNA_100338, circHIPK3, Hsa_circ_001569, hsa_circ_0005075, circ-PVT1, circ-10720, circRNA101368, circ_001569, has_circ_0078710, and circ-ZEB1.33 were upregulated, whereas hsa_circ_0001445, circSMAD2, Hsa_circ_0001649, hsa_circ_0005986, CircC3P1, hsa_circ_0004018, cirZKSCAN1, cSMARCA5, hsa_circ_0003570, hsa_circ_0068669, and hsa_circ_0064428 were downregulated. Altered circRNAs expression was significantly associated with tumor stage, differentiation, size, numbers, vascular invasion, organ metastasis, and AFP (alpha-fetoprotein) in almost studies. They might play crucial roles in tumorigenesis and tumor progression of HCC. Additionally, some studies also showed a relationship of circRNAs expression with liver cirrhosis or chronic hepatitis B.

## 4. Diagnostic Meta-Analysis

### 4.1. Study Characteristics and Quality Assessment

The baseline characteristics of the eligible studies were summarized in Table 2. Ten studies from 8 articles with 712 cases and 811 controls were included. All of the studies were published from 2016 to 2018 and conducted in China. Most of patients were male and pathologically diagnosed with HBV-associated HCC. The quantitative reverse transcription polymerase chain reaction (qRT-PCR) was used to measure the expression of 8 circRNAs, and the most common reference gene was GAPDH. In addition, specimens contain plasma and tissue. The quality of the studies was moderate. Further details of the quality assessment were summarized in Supplement Figure S1.

### 4.2. Pooled Diagnostic Performance

**Figure 2:**
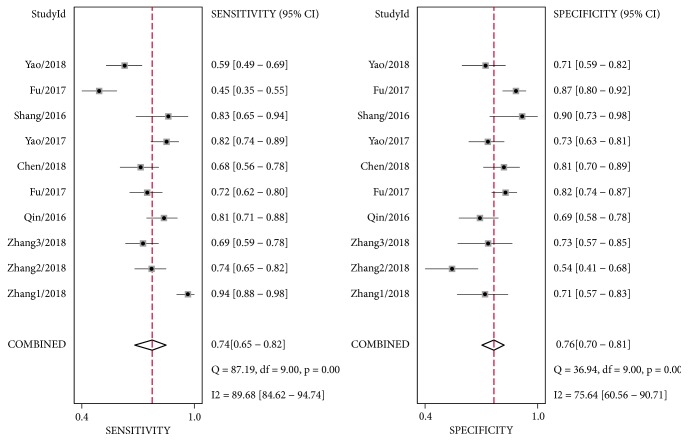
Forest plots of sensitivities and specificities of cirRNAs for the diagnosis of HCC.

**Figure 3:**
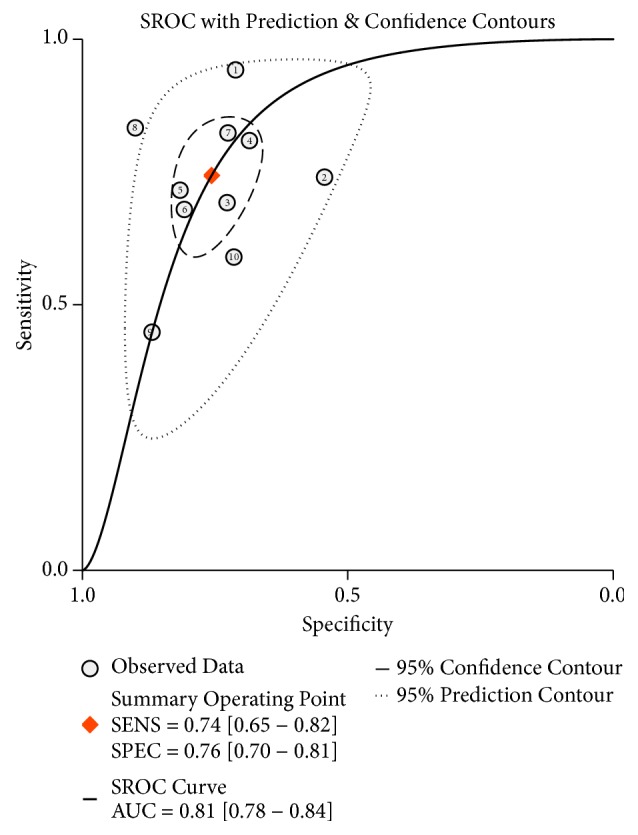
Summary receiver operating characteristic (SROC) curve of cirRNAs in the diagnosis of HCC.

As shown in Figure 2, considerable heterogeneity was observed among these studies (*I*^2^= 89.68%,* P* < 0.01 for sensitivity;* I*^2^= 75.64%,* P *< 0.01 for specificity). Therefore, a random-effect model was conducted. The pooled sensitivity, specificity, PLR, NLR, and DOR were 0.74 (95%CI: 0.65-0.82), 0.76 (95%CI: 0.70-0.81), 3.1 (95%CI: 2.50-3.80), 0.34 (95%CI: 0.25-0.47), and 9 (95%CI: 6 - 14), respectively. Moreover, the summary receiver operating characteristic curve (SROC) was also performed and the AUC was 0.81 (95% CI: 0.78-0.84) (Figure 3), indicating circRNAs had potential diagnostic value for HCC. In this study, threshold effect, the important source of heterogeneity, was explored. The spearman correlation coefficient was 0.248 (*P*=0.489), indicating no obvious threshold effect existed within included studies.

### 4.3. Subgroup Analysis and Meta-Regression Analysis

To explore the potential sources of heterogeneity, metaregression, and subgroup analyses were conducted according to the sample size, source of control, specimen type, reference gene, and male ratio. As presented in Table 3, circRNAs could more efficiently discriminate HCC from healthy individuals or adjacent nontumor tissues than from benign diseases (sensitivity: 0.83 versus 0.64, DOR: 14 versus 6, and AUC: 0.81 versus 0.75), and the heterogeneity reduced significantly from 71.4% to 56.7% and 55.6%, respectively. For the subgroup based on specimen type, plasma circRNAs might obtain a higher sensitivity and lower specificity (0.79 versus 0.68 and 0.65 versus 0.79, respectively). In addition, compared with the overall results, there were no significant differences in the studies with male (≥80%) or with GAPDH as a reference gene. According to the results of metaregression, none of these covariates above was responsible for the heterogeneity among included studies (*p *> 0.05). However, the source of control (RDOR: 2.65, 95% CI: 0.94-7.46,* P* = 0.06) might partially explain the heterogeneity.

### 4.4. Publication Bias and Sensitivity Analysis

**Figure 4:**
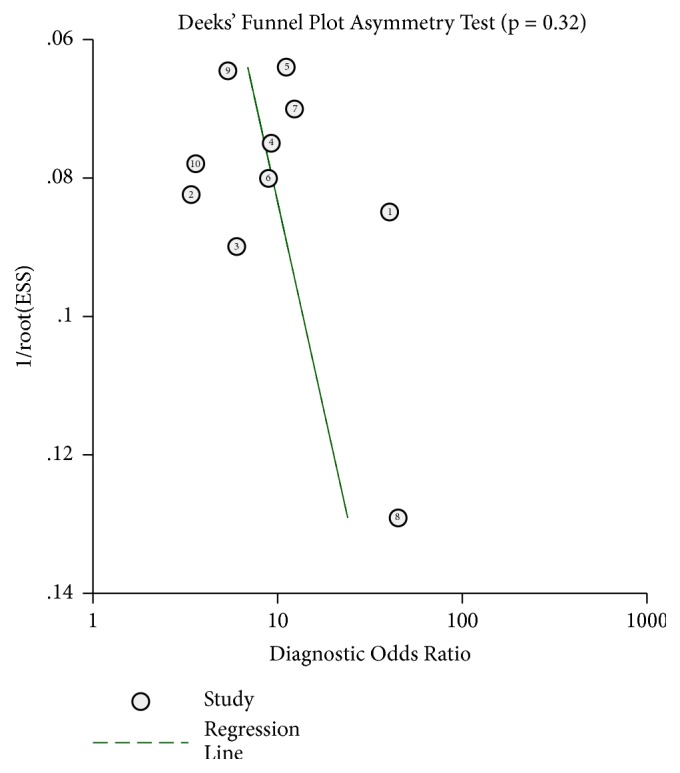
Deek's funnel plot to evaluate the publication bias of test accuracy.

**Figure 5:**
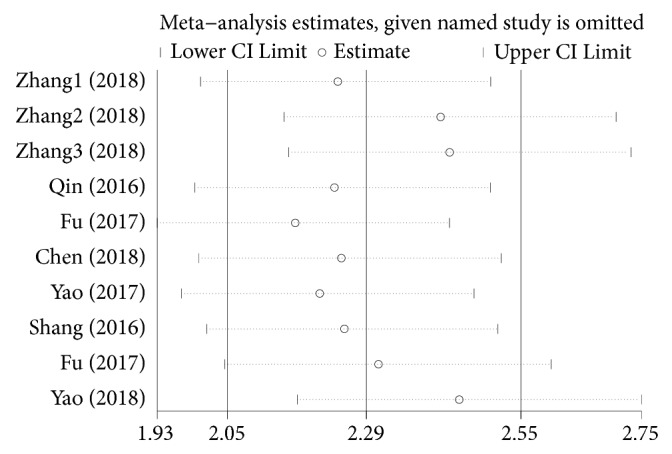
Sensitivity analysis of the overall pooled diagnostic studies (outlier detection analysis).

To evaluate the publication bias of the included studies, Deeks' funnel plot asymmetry test was performed. As indicated in Figure 4, a* P* value of 0.32 suggested that there was no significant publication bias. Sensitivity analysis was further performed. As displayed in Figure 5, the results were stable and not significantly affected by any individual study.

## 5. Prognostic Meta-Analysis

### 5.1. Study Characteristics and Quality Assessment

As present in Table 4, 12 studies from 11 articles with 1185 cases were included in this prognosis analysis. All these studies were conducted in China and published from 2016 to 2018. The qRT-PCR and FISH were adopted to quantify the level of circRNAs in tissues with GAPDH and b-actin as reference genes. OS and RFS were used to evaluate the outcome of the cohorts. A total of 11 different circRNAs were investigated. Increased expression of hsa_circ_0128298, ciRS-7, circRNA101368, circ_001569, and circRNA_100338 and decreased expression of CircC3P1, cSMARCA5, hsa_circ_0001649, hsa_circ_0064428, circ-ITCH, and circMTO1 were associated with worse prognosis. HRs and 95% CI were directly reported in 7 studies, and the remaining were extrapolated and calculated from Kaplan-Meier curves. The NOS scores varied from 5 to 7, suggesting that the quality of included studies was moderate. Details of quality assessment were present in Supplement Table S1.

### 5.2. Association between circRNAs and Outcomes

**Figure 6:**
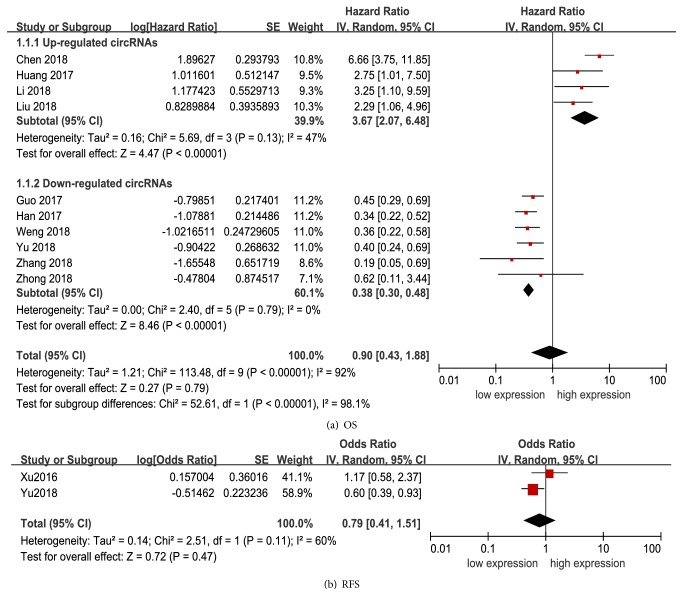
Forest plot for the association between altered cirRNAs expression and survival in HCC. (a) Association with overall survival; (b) association with recurrence free survival. SE, standard error; IV, inverse variance methods; HR, hazard ratio; CI, confidence interval.

Due to significant heterogeneity among studies existed (*I*^2^ = 92%,* P* < 0.01), a random-effects model was performed. As shown in Figure 6, the pooled HR of OS was 0.90 (95%CI: 0.43-1.88) for high versus low cirRNAs expression. Stratified analysis according to altered expression was then performed. The pooled HR for upregulated circRNAs and downregulated circRNAs were 3.67 (2.07-6.48) and 0.38 (0.30-0.48), respectively (Figure 6(a)), and the heterogeneity reduced significantly from 92% to 47% and 0%, respectively. The increased expression of upregulated circRNAs or decreased expression of downregulated circRNAs was significantly related to a worse prognosis. Metaregression analysis for this subgroup suggested that altered expression was the main source of heterogeneity (*P* < 0.01).

Additionally, two studies including 258 patients reported HRs for RFS. The overall result revealed that circRNAs expression was not associated with RFS in HCC patients (HR: 0.79, 95%CI: 0.41-1.51,* P* = 0.47) (Figure 6(b)).

### 5.3. Publication Bias

**Figure 7:**
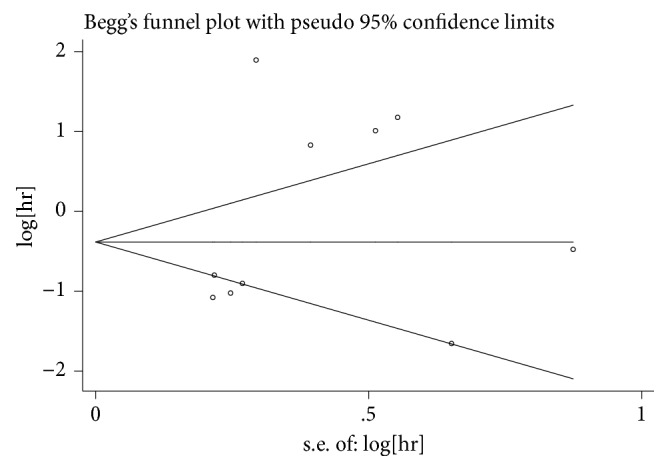
Begg's funnel plots for all of the included studies reported with overall survival.

Publication bias was checked by Begg's funnel plot. As suggested in Figure 7, a* P* value of 0.19 suggested that there was no significant publication bias among these studies.

## 6. Discussion

Increasing studies have demonstrated that circRNAs are relatively stable and detectable in body fluids and tissues and may serve as promising biomarkers for cancer diagnosis and prognosis [46]. Herein, we implemented this comprehensive review to evaluate the clinical value of circRNAs for HCC. According to the results of this study, the diagnostic accuracy of circRNAs for HCC was moderate. Moreover, altered circRNAs expression was significantly associated with tumor characteristic, and increased expression of upregulated circRNAs or decreased expression of downregulated circRNAs could predict worse OS in HCC patients. These findings indicate that circRNAs may serve as promising biomarkers for HCC diagnosis and prognosis prediction.

For diagnostic value, four previous meta-analyses concluded that the overall sensitivity, specificity, and NLR of circRNA for HCC were from 0.73 to 0.82, 0.72 to 0.79, and 0.34, with PLR ranging from 3.40 to 3.51, DOR from 10.00 to 10.21, and AUC from 0.83 to 0.86, respectively [10–13]. Consistent with these findings, in our study, the pooled sensitivity, specificity, and AUC of circRNAs were 0.74, 0.76, and 0.81, respectively, indicating a moderate diagnostic accuracy. The pooled DOR, a global measure of diagnostic performance [47], was 9, suggesting that circRNAs could effectively discriminate HCC patients from noncancerous controls. The pooled PLR was 3.1, suggesting that there was 3.1-fold higher possibility of altered expression of circRNAs for patients with HCC compared to those without. Likewise, NLR of 0.34 indicates that people with normal expression of circRNAs still have a 34% chance of having HCC. In stratified analysis, expectedly, the diagnostic value of circRNAs varied according to the source of control. CircRNAs could more efficiently discriminate HCC from healthy individuals than from benign diseases. Furthermore, in accordance with previous studies [48–50], the characteristics of detection methods may also affect the diagnostic performance of circRNAs. Plasma circRNAs might obtain higher sensitivity and lower specificity in this study. Therefore, standardized protocol needs to be established to minimize protocol-based bias, and make the results more comparable.

Although the diagnostic performance of circRNAs was not good enough to confirm or exclude the diagnosis of HCC, circRNAs still have great advantages over the traditional clinical marker, and when combined with other biomarkers or clinical examinations, circRNAs may obtain a better diagnostic performance. As Fu et al. reported, hsa_circ_0004018 is a valuable biomarker for HCC diagnosis, with its superior sensitivity to alpha-fetoprotein (AFP) [25]. Similarly, Plasma hsa_circ_0001445 also had a higher diagnostic accuracy than AFP for distinguishing HCC patients from healthy people or patients with hepatitis B. And when combined, the efficiency in distinguishing HCC from healthy controls (AUC: 0.970, 95% CI: 0.949–0.991), from cases of cirrhosis (AUC: 0.743, 95% CI: 0.664–0.821), or from cases of hepatitis B (AUC: 0.877, 95% CI: 0.817–0.938) was higher [22]. However, further multicenter and high-quality studies are still required to explore their diagnostic value as promising biomarkers.

Advanced studies have revealed that plenty of circRNAs are differentially expressed in HCC. They play crucial roles in tumorigenesis and tumor progression [6, 7, 51] and are significantly correlated with clinicopathological features, especially tumor characteristic. For instance, circSMAD2 inhibits the migration, invasion, and EMT of HCC cells by targeting miR-629 [23] and markedly associates with the differentiation degree; circC3P1 acts as a tumor suppressor via enhancing PCK1 expression by sponging miR-4641 to inhibit HCC growth and metastasis. It negatively correlated with TNM stage, tumor size, and vascular invasion and might serve as a prognostic biomarker [34]. In addition, Hsa_circ_0005986 also functioned as microRNA sponge in tumorigenesis and accelerated cell proliferation by promoting the G0/ G1 to S phase transition in liver cancer cells and was correlated with tumor diameters, stage, and microvascular invasion [24]. All these evidences suggest that circRNAs may be risk factors for the outcome in HCC patients.

However, currently, it remains controversial whether circRNAs could serve as prognostic markers. Zhang et al. reported that hsa_circ_0001649 expression was a novel independent prognostic factor for a better OS of HCC patients [35], while upregulated circRNA_100338 closely correlated with a lower cumulative survival rate and metastatic progression in HCC patients with hepatitis B [28]. Moreover, elevated ciRS-7 expression in HCC showed a shorter time of tumor recurrence than that of patients with decreased ciRS-7 expression, but no statistical significance was observed [45]. In this meta-analysis, we found that upregulated or downregulated circRNAs were significantly associated with OS in HCC, and the predictive efficacy was significant, suggesting a value of employing circRNAs as biomarkers for HCC prognosis, which were consistent with previous meta-analysis and studies [13]. Regrettably, due to limited studies (n=2), we failed to draw clear conclusions on the association between circRNAs and RFS in HCC patients. Therefore, further large-scale investigations are demanded to comprehensively and objectively investigate their promising prognostic value for HCC.

Compared with previous meta-analyses [10–13], we included more diagnostic studies, which would make our assessment more precise. What is more, further detailed analyses of diagnostic value of circRNAs for HCC were performed according to the sample size, source of control, specimen type, reference gene, and male ratio. In addition, the association between circRNAs expression and survival of HCC patients (OS and RFS) were also comprehensively evaluated. However, the following limitations merit consideration. Firstly, the number of studies and sample size are still relatively small, so our findings needed more large cohorts to validate. Secondly, due to unavailable original data, we failed to quantificationally evaluate the association of circRNAs with clinicopathological features and make more confirming conclusions. Thirdly, several HRs could not be directly extracted from 4 studies and were calculated from the Kaplan-Meier survival curves, which might be less reliable and biased our results. Fourthly, obvious heterogeneity existed across the included studies. The source of control and altered expression might be the sources of heterogeneity in diagnostic and prognostic meta-analysis, respectively. However, we failed to find other potential sources. Lastly, all included studies were conducted in China; therefore, our conclusions might not be universally suitable.

In summary, our meta-analysis indicates that aberrant circRNAs expression closely correlated with the clinicopathological characteristics of HCC, and it is possible that these circRNAs may serve as promising diagnostic and prognostic biomarkers for HCC. However, due to the limitations of this meta-analysis, well-designed, larger-size, and higher-quality prospective studies are required to confirm the clinical value of circRNAs as tumor markers and draw more definitive conclusions.

## Figures and Tables

**Table 1:** Altered circRNAs expression associated with clinicopathological features of HCC in 23 eligible articles.

Study	Sample size	circRNA	Altered expression	Test Method	Specimen	Co-variants (clinicopathological parameters)	Function	Reference
Zhang 2018	73/104	hsa_circ_0001445	Down	qRT-PCR	Tissue	Tissue: Tumor foci; Spasm: AFP	Apoptosis/proliferation/ migration/invasion	[22]
					Spasm			
Zhang 2018	86	circSMAD2	Down	qRT-PCR	Tissue	Differentiation	Migration/invasion/ EMT	[23]
Qin 2016	89	Hsa_circ_0001649	Down	qRT-PCR	Tissue	tumor size and embolus	Metastasis	[8]
Fu 2017	81	Hsa_circ_0005986	Down	qRT-PCR	Tissue	BCLC stage, chronic hepatitis B, tumor size, MVI	Carcinogenesis	[24]
Fu 2017	102	hsa_circ_0004018	Down	qRT-PCR	Tissue	AFP, tumor size, differentiation, BCLC and TNM stage	Carcinogenesis/metastasis	[25]
Chen 2018	78	hsa_circ_0128298	Up	qRT-PCR	Tissue	vascular cancer embolus, lymphatic metastasis and organ metastasis	Proliferation/metastasis	[26]
Yao 2017	102	cirZKSCAN1	Down	qRT-PCR	Tissue	tumor number, liver cirrhosis, vascular invasion and TNM stage	Growth/migration/invasion	[27]
Huang 2017	80	circRNA_100338	Up	qRT-PCR	Tissue	Vascular invasion, Lung metastasis and TNM stage	Metastasis	[28]
Chen 2018	50	circHIPK3	Up	qRT-PCR	Tissue	TNM stage, differentiation, HBV-DNA copy numbers and liver cirrhosis	Proliferation/migration	[29]
Jin 2017	30	Hsa_circ_001569	Up	qRT-PCR	Tissue	tumor differentiation and TNM stage	Proliferation/ growth	[30]
Shang 2016	30	hsa_circ_0005075	Up	qRT-PCR	Tissue	tumor size	Proliferation/invasion/ metastasis	[31]
Yu 2018	163	cSMARCA5	Down	qRT-PCR	Tissue	Tumor size, differentiation, TNM stage, BCLC stage, Edmondson's grade and MVI	Growth/metastasis	[9]
Fu 2018	107	hsa_circ_0003570	Down	qRT-PCR	Tissue	tumor size, differentiation, AFP, MVI, BCLC stage and TNM stage	Recurrence/metastasis	[32]
Yao 2018	100	hsa_circ_0068669	Down	qRT-PCR	Tissue	MVI and TMN stage	Progression	[33]
Zhong 2018	47	CircC3P1	Down	qRT-PCR	Tissue	TNM stage, tumor size and vascular invasion	Proliferation/migration/ invasion	[34]
Zhang 2018	77	hsa_circ_0001649	Down	qRT-PCR	Tissue	No significant correlation	Apoptosis/proliferation/ migration/invasion	[35]
Zhu 2018	46	circ-PV T1	Up	qRT-PCR	Tissue	Tumor size, differentiation and TNM stage	Proliferation	[36]
Meng 2018	75	circ-10720	Up	FISH	Tissue	Tumor stage, AFP and HBV markers	Development/progression	[37]
Li 2018	51	circRNA101368	Up	qRT-PCR	Tissue	Tumor size, distant metastasis and TNM stage	Migration	[38]
Liu 2018	70	circ_001569	Up	qRT-PCR	Tissue	Tumor size and TNM stage	Growth/metastasis	[39]
Weng 2018	120	hsa_circ_0064428	Down	qRT-PCR	Tissue	Tumor size, differentiation and TNM stage	Tumourigenesis/metastasis	[40]
Xie 2018	56	has_circ_0078710	Up	qRT-PCR	Tissue	TNM stage	Proliferation/migration/ invasion/growth	[41]
Gong 2018	64	circ-ZEB1.33	Up	qRT-PCR	Tissue	Tumor size and TNM stage	Proliferation	[42]
					serum			

EMT: epithelial-mesenchymal transition; BCLC: Barcelona Clinic Liver Cancer Staging System; AFP: alpha-fetoprotein; TNM: tumor-node-metastasis; MVI: microvascular invasion; FISH: fluorescence in situ hybridization; HBV: hepatitis B virus.

**Table 2:** Main characteristics of 10 studies included in diagnostic meta-analysis.

Author, year	Country	Sample size (case/control)	Male (case)	HBsAg (case)	Tumor size (≤5)	Control	Profile	Specimen type	Method	Reference gene	Sensitivity (%)	Specificity (%)
Zhang et al. 2017	China	104/52	87	NA	NA	healthy people	hsa_circ_0001445	plasma	qRT-PCR (SYBR)	GAPDH	94.20	71.20
		104/57	87	NA	NA	liver cirrhosis	hsa_circ_0001445	plasma	qRT-PCR (SYBR)	GAPDH	74.00	54.40
		104/44	87	NA	NA	chronic hepatitis B	hsa_circ_0001445	plasma	qRT-PCR (SYBR)	GAPDH	69.20	72.70
Qin et al. 2016	China	89/89	74	66	50	adjacent non-tumor tissues	hsa_circ_0001649	tissue	qRT-PCR (SYBR)	*β*-actin	81.00	69.00
Zhang et al. 2017	China	102/152	90	86	62	adjacent non-tumor tissues and chronic hepatitis	hsa_circ_0004018	tissue	qRT-PCR (SYBR)	GAPDH	71.60	81.50
Chen et al. 2018	China	78/78	70	65	NA	adjacent non-tumor tissues	hsa_circ_0128298	tissue	qRT-PCR (SYBR)	GAPDH	67.40	80.50
Yao et al. 2017	China	102/102	87	85	29	adjacent non-tumor tissues	cirZKSCAN1	tissue	qRT-PCR (SYBR)	GAPDH	82.20	72.40
Shang et al. 2016	China	30/30	25	23	19	adjacent non-tumor tissues	hsa_circ_0005075	tissue	qRT-PCR (SYBR)	GAPDH	83.30	90.00
Fu et al. 2017	China	107/137	96	90	62	chronic hepatitis and liver cirrhosis	hsa_circ_0003570	tissue	qRT-PCR (SYBR)	GAPDH	44.90	86.80
Yao et al. 2018	China	100/70	14	NA	61	chronic hepatitis	hsa_circ_0068669	tissue	qRT-PCR (SYBR)	GAPDH	59.00	71.00

HBsAg: surface antigen of the hepatitis B virus; qRT-PCR: quantitative reverse transcription PCR; NA: not available.

**Table 3:** Results of subgroup analysis and univariate metaregression in diagnostic meta-analysis.

Covariate	No. of studies	Heterogeneity	Sensitivity	Specificity	DOR	AUC	RDOR	*P *
		*I* ^2^ test (%)/*P*_*h*_	(95%CI)	(95%CI)	(95%CI)	(95%CI)	(95%CI)	
*Sample size*			.					
N ≥170	5	60.40/ 0.04	0.69 (0.55 - 0.80)	0.78 (0.71 - 0.83)	8.00 (5.00 - 12.00)	0.81 (0.77 - 0.84)	0.83 (0.23-3.03)	0.74
N <170	5	81.10/<0.01	0.80 (0.67 - 0.88)	0.74 (0.63 - 0.83)	11.00 (5.00 - 27.00)	0.83 (0.80 - 0.86)		
*Source of control*								
Healthy people or adjacent non-tumor tissues	5	56.70/ 0.06	0.83 (0.73 - 0.90)	0.75 (0.69 - 0.80)	14.00 (9.00 - 23.00)	0.81 (0.78 - 0.84)	2.65 (0.94-7.46)	*0.06*
Liver cirrhosis or chronic hepatitis	5	55.60/0.06	0.64 (0.54 - 0.73)	0.75 (0.64 - 0.84)	6.00 (4.00 – 8.00)	0.75 (0.71 - 0.79)		
*Specimen type*								
Plasmas	3	87.30/<0.01	0.79 (0.74 - 0.84)	0.65 (0.57 - 0.73)	8.93 (2.37 – 33.64)	0.72 (0.45 - 1.00)	0.57 (0.13-2.56)	0.41
Tissue	7	60.50/ 0.02	0.68 (0.64 - 0.72)	0.79 (0.75 - 0.82)	8.62 (5.58 – 13.30)	0.82 (0.77 - 0.87)		
*Reference gene*	9	74.30/<0.01	0.74 (0.63 - 0.82)	0.76 (0.70 - 0.82)	9.00 (5.00 - 15.00)	0.82 (0.78 - 0.85)		
(GAPDH)								
*Male* (≥80%)	9	68.40/<0.01	0.76 (0.66 - 0.84)	0.76 (0.69 - 0.82)	10.00 (6.00 – 16.00)	0.82 (0.79 - 0.85)		
*Overall*	10	71.40/<0.01	0.74 (0.65 - 0.82)	0.76 (0.70 - 0.81)	9.00 (6.00 – 14.00)	0.81 (0.78 - 0.84)		

CI: confidence interval; DOR: diagnostic odds ratio; RDOR: Relative diagnostic odds ratio; AUC: the area under the SROC curve.

**Table 4:** Main characteristics of 11 articles included in prognostic meta-analysis.

Author, year	Country	Case	circRNAs	Altered expression	Specimen type	Method	Reference gene	Outcome	Follow-up (month)	Expression related to poor prognosis	HR (95%CI)	Analysis method	NOS (scores)
Chen et al. 2018	China	78	hsa_circ_0128298	Up	tissue	qRT-PCR (SYBR)	GAPDH	OS	~67	Up	6.661 (2.661-8.418)	Multivariate	6
Huang et al. 2017	China	80	circRNA_100338	Up	tissue	qRT-PCR (SYBR)	GAPDH	OS	~120	Up	2.75 (1.01-7.52)	Kaplan-Meier curves	7
Yu et al. 2018	China	163	cSMARCA5	down	tissue	qRT-PCR (SYBR)	GAPDH	OS RFS	~60	down	2.47 (1.459-4.182) 1.673 (1.08-2.591)	Multivariate	7
Xu et al. 2017	China	95	ciRS-7	Up	tissue	qRT-PCR (SYBR)	GAPDH	RFS	~63	Up	1.17 (0.58 - 2.38)	Multivariate	7
Zhong et al. 2018	China	47	CircC3P1	down	tissue	qRT-PCR (SYBR)	b-actin	OS	~60	down	0.62 (0.11 - 3.39)	Kaplan-Meier curves	6
Zhang et al. 2018	China	77	hsa_circ_0001649	down	tissue	qRT-PCR (SYBR)	GAPDH	OS	~45	down	0.191 (0.053-0.682)	Multivariate	7
Guo et al. 2017	China	288	circ-ITCH	down	tissue	qRT-PCR (SYBR)	GAPDH	OS	~83	down	0.45 (0.29 - 0.68)	Multivariate	6
Han et al. 2017	China	116	circMTO1	down	tissue	FISH	-	OS	~80	down	0.34 (0.22 - 0.51)	Kaplan-Meier curves	5
Li et al. 2018	China	51	circRNA101368	Up	tissue	qRT-PCR (SYBR)	GAPDH	OS	~60	Up	3.246 (1.098-9.594)	Multivariate	6
Liu et al. 2018	China	70	circ_001569	Up	tissue	qRT-PCR (SYBR)	NA	OS	~60	Up	2.291 (1.059-4.954)	Multivariate	7
Weng et al. 2018	China	120	hsa_circ_0064428	down	tissue	qRT-PCR (SYBR)	NA	OS	~72	down	0.36 (0.22-0.58)	Kaplan-Meier curves	7

qRT-PCR: quantitative reverse transcription PCR; FISH: fluorescence in situ hybridization; OS: overall survival; RFS: recurrence free survival; NA: not available; HR: hazard ratio; NOS: the Newcastle-Ottawa scale.

## Abbreviations

circRNAs: Circular RNAs
HCC: Hepatocellular carcinoma
qRT-PCR: Quantitative reverse transcription PCR
DOR: Diagnostic odds ratio
LR: Likelihood ratio
AUC: Area under the SROC
SROC: Summary receiver operator characteristic curve
QUADAS-2: Quality Assessment of Diagnostic Accuracy Studies 2
OS: Overall survival
RFS: Recurrence free survival
HR: Hazard ratio
NOS: Newcastle-Ottawa Scale.

## Supplementary Materials

Supplementary 1 File S1. PRISMA 2009 Checklist (DOC).

Supplementary 2 Figure S1. Details of quality assessment by the QUADAS-2 tool in diagnosis meta-analysis. “-” in red, “?” in yellow, and “+” in green mean high risk, unclear risk, and low risk, respectively.

Supplementary 3 Table S1. Assessment of the risk of bias in each cohort study with the Newcastle-Ottawa Scale in prognosis meta-analysis (DOC).
