# Supplementary figures and images for: Systematic Review and Meta-Analysis of the Utility of Circular RNAs as Biomarkers of Hepatocellular Carcinoma
Source: Can J Gastroenterol Hepatol. 2019 May 2;2019:1684039. doi: 10.1155/2019/1684039 (PMC6521581; doi:10.1155/2019/1684039)

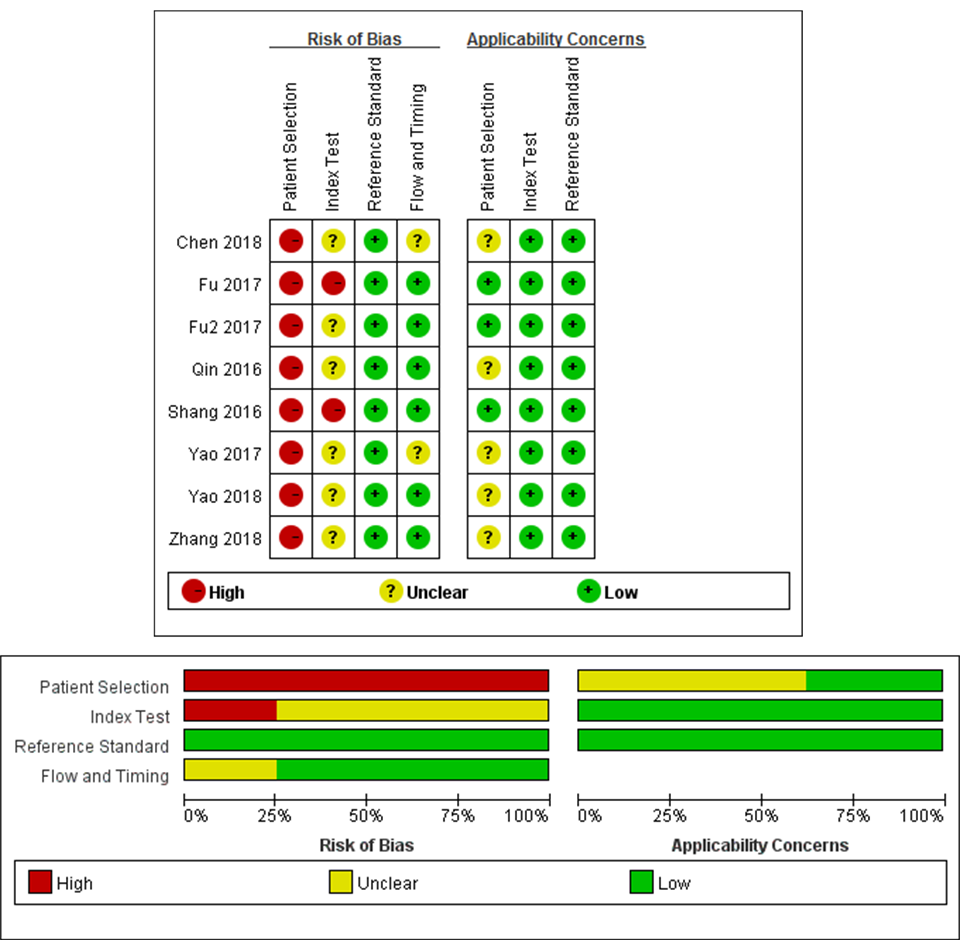

Supplement: Supplementary 2 — Figure S1. Details of quality assessment by the QUADAS-2 tool in diagnosis meta-analysis. “-” in red, “?” in yellow, and “+” in green mean high risk, unclear risk, and low risk, respectively. [file 1684039.f2.tif]
