# Supplementary material for: Systematic Review and Meta-Analysis of the Utility of Circular RNAs as Biomarkers of Hepatocellular Carcinoma
Source: Can J Gastroenterol Hepatol. 2019 May 2;2019:1684039. doi: 10.1155/2019/1684039 (PMC6521581; doi:10.1155/2019/1684039)
Supplement: Supplementary 3 — Table S1. Assessment of the risk of bias in each cohort study with the Newcastle-Ottawa Scale in prognosis meta-analysis (DOC). [file 1684039.f3.doc]

**Table S1 Assessment of the risk of bias in each cohort study with the Newcastle-Ottawa scale in prognosis meta-analysis**

| **Study** | **Selection** | | | | **Comparability** | **Outcome** | | | **Score** |
| --- | --- | --- | --- | --- | --- | --- | --- | --- | --- |
|  | **1 Representativeness**  **score of exposed cohort** | **2 Selection of unexposed cohort** | **3 Ascertainment**  **of exposure** | **4 Outcome**  **of interest** | **5 Comparability of cohorts** | **6** **Assessment of outcome** | **7 Length of follow-up** | **8 Adequacy of follow-up** |  |
| Chen et al. 2018 | 0 | 0 | 1 | 0 | 2 | 1 | 1 | 1 | 6 |
| Huang et al. 2017 | 1 | 1 | 1 | 0 | 1 | 1 | 1 | 1 | 7 |
| Yu et al. 2018 | 0 | 1 | 1 | 0 | 2 | 1 | 1 | 1 | 7 |
| Xu et al. 2017 | 0 | 1 | 1 | 0 | 2 | 1 | 1 | 1 | 7 |
| Zhong et al. 2018 | 0 | 1 | 1 | 0 | 1 | 1 | 1 | 1 | 6 |
| Zhang et al. 2018 | 1 | 1 | 1 | 0 | 2 | 1 | 0 | 1 | 7 |
| Guo et al. 2017 | 0 | 1 | 1 | 0 | 1 | 1 | 1 | 1 | 6 |
| Han et al. 2017 | 0 | 1 | 1 | 0 | 0 | 1 | 1 | 1 | 5 |
| Li et al. 2018 | 0 | 1 | 1 | 0 | 1 | 1 | 1 | 1 | 6 |
| Liu et al.  2018 | 0 | 1 | 1 | 0 | 2 | 1 | 1 | 1 | 7 |
| Weng et al. 2018 | 0 | 1 | 1 | 0 | 2 | 1 | 1 | 1 | 7 |

**Abbreviations:** “1” indicates that the study has satisfied the item and “0” indications the opposite
1. The exposed cohort truly or somewhat represented the average in the community.
2. The unexposed cohort was drawn from the same community as the exposed cohort.
3. Ascertainment of exposure was secure record or structured interview.
4. Outcome of interest was not present at start of study.
5. Study controls for the most important factor (sex, year, other confounding factors).
6. Assessment of outcome was from independent blind assessment or record linkage.
7. Follow‑up was long enough for outcomes to occur (≥60 months).
8. No subject lost to follow‑up or subjects lost to follow‑up unlikely to introduce bias or description provided of those lost (≥90%)
